# Effects of Carboxymethylcellulose Artificial Tears on Ocular Surface Microbiome Diversity and Composition, A Randomized Controlled Trial

**DOI:** 10.1167/tvst.12.8.5

**Published:** 2023-08-09

**Authors:** Yujia Zhou, Gurjit S. Sidhu, Joan A. Whitlock, Bishoy Abdelmalik, Zachary Mayer, Youlei Li, Gary P. Wang, Walter A. Steigleman

**Affiliations:** 1 Department of Ophthalmology, University of Florida Shands Hospital, Gainesville, FL, USA; 2 Division of Infectious Diseases and Global Medicine, Department of Medicine, University of Florida, Gainesville, FL, USA; 3 University of Florida College of Medicine, Gainesville, FL, USA

**Keywords:** microbiome, bacterial, dry eye, artificial tear, infection

## Abstract

**Purpose:**

Carboxymethylcellulose is an artificial tear ingredient known to decrease gut microbiome diversity when ingested. This study examines the effect of carboxymethylcellulose on ocular surface microbiome diversity and composition.

**Methods:**

Healthy adult participants without significant ophthalmic disease or concurrent carboxymethylcellulose artificial tear use were allocated randomly to take carboxymethylcellulose or control polyethylene glycol artificial tears for seven days. Conjunctival swabs were collected before and after artificial tear treatment. This trial is registered at clinicaltrials.gov (NCT05292755). Primary outcomes included abundance of bacterial taxa and microbiome diversity as measured by the Chao-1 richness estimate, Shannon's phylogenetic diversity index, and UniFrac analysis. Secondary outcomes included Ocular Surface Disease Index scores and artificial tear compliance.

**Results:**

Of the 80 enrolled participants, 66 completed the trial. Neither intervention affected Chao-1 richness (analysis of variance [ANOVA], *P* = 0.231) or Shannon's diversity index (ANOVA, *P* = 0.224). Microbiome samples did not separate by time point (permutation multivariate analysis of variance [PERMANOVA], *P* = 0.223) or intervention group (PERMANOVA, *P* = 0.668). LEfSe taxonomic analysis revealed that carboxymethylcellulose depleted several taxa including *Bacteroides* and *Lachnoclostridium*, but enriched *Enterobacteriaceae*, *Citrobacter*, and *Gordonia*. Both interventions decreased OSDI scores (Wilcoxon signed rank test, *P* < 0.05), but there was no significant difference between interventions (Mann-Whitney *U*, *P* = 0.54).

**Conclusions:**

Carboxymethylcellulose artificial tears increased *Actinobacteriota* but decreased *Bacteroides* and *Firmicutes* bacteria. Carboxymethylcellulose artificial tears do not affect ocular surface microbiome diversity and are not significantly more effective than polyethylene glycol artificial tears for dry eye treatment.

**Translational Relevance:**

The 16S microbiome analysis has revealed small changes in the ocular surface microbiome associated with artificial tear use.

## Introduction

Dry eye syndrome (DES) is a disorder of the ocular surface, frequently characterized by lipid or aqueous deficiency of the tear film.^1^^,^^2^ Associated symptoms such as stinging, burning, itching, light sensitivity, and blurry vision may reduce patient quality of life and productivity.^3^ In chronic cases, the ocular surface including the tear film, cornea, and conjunctiva may develop lasting structural damage.^4^^,^^5^ Mounting evidence now suggests that the disorder is multifactorial, involving neurotrophic deficiency, meibomian gland dysfunction, and inflammation. Antigen-presenting cells and T helper 17 cells on the ocular surface may mediate ocular surface inflammation.^6^ Indeed, several immunomodulatory therapeutics are effective treatments for DES, although factors inciting inflammation in DES are not definitive.^7^

One potential factor mediating the inflammatory pathophysiology of DES is the ocular surface microbiome (OSM), composed of all microbial flora colonizing the ocular surface.^8^ In the human OSM, *Corynebacterium, Staphylococcus, Streptococcus, Propionibacterium, Pseudomonas,* and *Acinetobacter* are the primary bacterial genera.^9^^–^^12^ Fungal and viral genera include the fungi *Aspergillus*, *Malassezia*, *Setosphaeria*, and *Haematonectria*, as well as the viruses *human* *papilloma* *virus* and *Torque* *teno* *virus*.^13^^–^^15^ Environmental factors and ocular diseases are related to OSM composition. For example, sex and ethnicity have not been associated with unique OSM features,^16^^,^^17^ whereas age, climate, and contact lens use are associated with changes in the OSM composition.^18^^–^^20^ It is therefore unsurprising that DES has been associated with increased microbial diversity and changes in species abundance.^21^^,^^22^

Artificial tears (ATs) are a mainstay of DES therapy.^23^^,^^24^ Formulations attempt to match the properties of endogenous tears and are produced with or without a preservative.^25^ They typically contain electrolytes and a thickener such as carboxymethylcellulose (CMC), hyaluronic acid, or polyethylene glycol (PEG). The thickener is crucial to AT retention on the ocular surface, as endogenous tears have higher viscosity than saline solution, which allows the tear film to resist washout from shear forces during blinks.^26^ Ingested CMC, however, has been shown to cause decreased gut microbiome diversity and modified abundance of bacterial genera such as *Faecalibacterium*, *Ruminococcus*, *Roseburia,* and *Lachnospiraceae*.^27^ Animal studies also suggest that these changes may result in clinically significant immunologic changes.^28^ Studies on the eyelid microbiome and OSM have shown that prostaglandin analogues increase beta diversity, topical anesthetics have been shown to decrease *Cutibacterium* species, and sodium hyaluronate have been shown to decrease abundance of specific bacterial species such as *Flavobacterium caeni* and *Deinococcus antarcticus*.^29^^–^^32^ It therefore stands to reason that CMC-ATs may also have measurable effects on the OSM.

Given the widespread use of CMC-ATs and its potential implications for the OSM, we conducted a double-masked randomized controlled trial examining the effects of CMC-ATs on the OSM and DES symptoms, compared to a control PEG-AT without CMC. Outcomes included metagenomic analysis of the OSM by 16S bacterial ribosomal RNA (rRNA) sequencing,^33^ subjective DES symptoms scored using the Ocular Surface Disease Index (OSDI) survey,^34^^,^^35^ and AT compliance surveys.

## Methods

### Patients and Study Design

**Figure 1.**
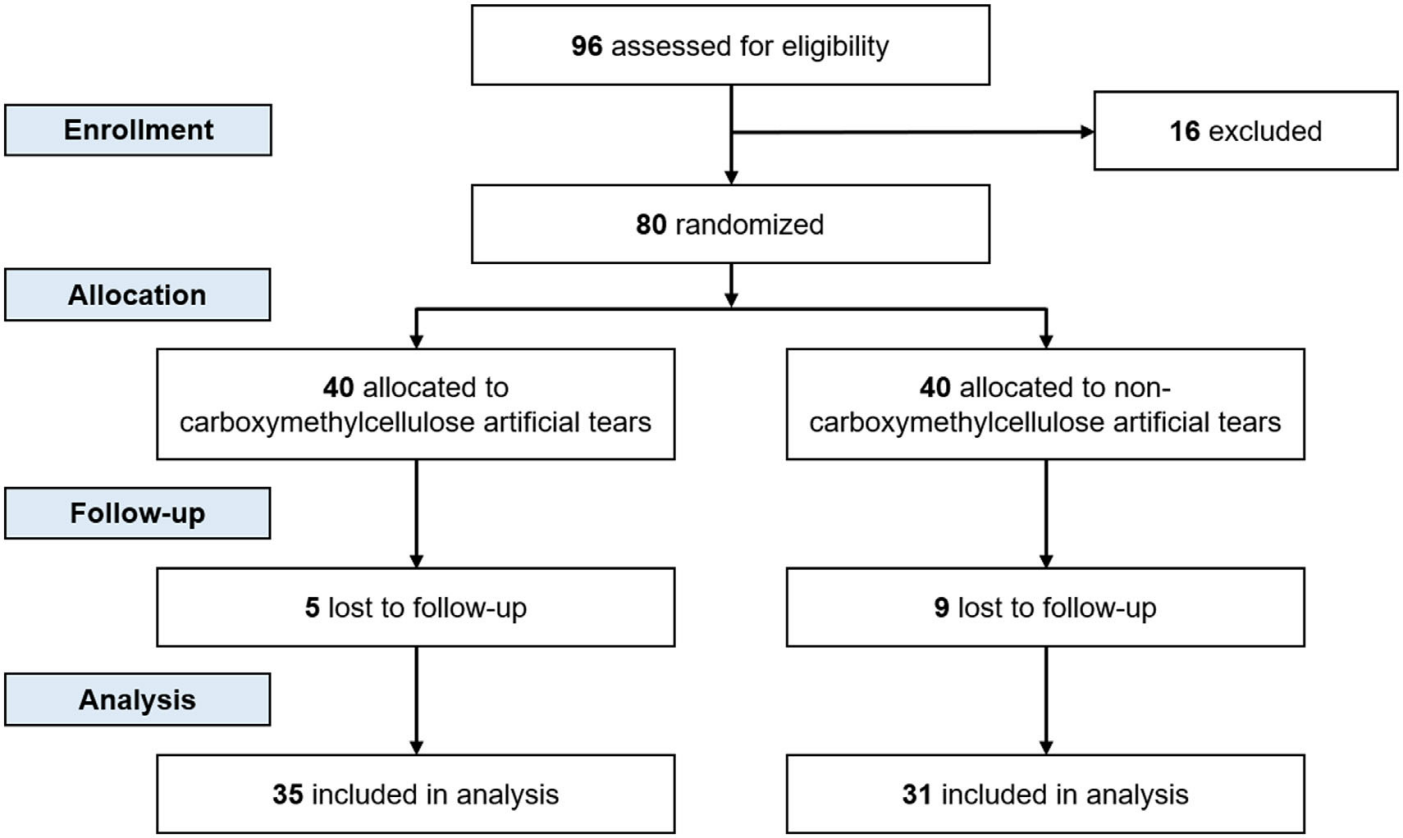
Study design and participant flow chart. After 96 prospective participants were assessed for eligibility, 80 participants were enrolled. Patients were assigned by simple randomization to receive either CMC or PEG ATs, masked to the participant and the researcher. Conjunctival swabs and surveys were administered on days 1 and 7. Participant dropout was five for the CMC group and nine for the PEG group.

This is a double-masked randomized controlled trial (Fig. 1) conducted at the University of Florida Oaks Eye Center from April 2022 to May 2022. Adults aged 18 years or older with the ability to self-administer preservative-free ATs were eligible for enrollment. Adults with active eye infections, ocular prostheses, autoimmune diseases, cancers of the eye, or history of CMC-AT use, immunomodulatory therapy, steroids, antibiotics, preserved eyedrops, and medicated eyedrops within one week of study enrollment were excluded. The trial was conducted in compliance with the Declaration of Helsinki and the Health Insurance Portability and Accountability Act. Institutional review board approval was obtained at the University of Florida, and informed consent was obtained from each patient before data collection. The trial is registered at https://clinicaltrials.gov/ct2/show/NCT05292755.

### Intervention and Outcomes

Enrolled participants were randomized to receive either ATs containing 0.5% CMC (Refresh Plus; Allergan Inc., Dublin, Ireland) or control ATs containing 0.4% PEG (Systane PF; Alcon Labs, Fort Worth, TX, USA). For the purposes of masking, one researcher with no participant contact (Li, Y.) created 80 identical boxes, each labeled with a unique alphanumeric code. Boxes contained 30 similar, unlabeled, and transparent vials of either CMC (n = 40) or PEG (n = 40) ATs. Each alphanumeric code and its contents were recorded on a spreadsheet (Excel 2016; Microsoft, Redmond, WA, USA) and randomly paired with a numerical code (1–80) using a computer algorithm. Required sample size was calculated by power estimates in IBM SPSS 28 (IBM Corp., Armonk, NY, USA) with anticipated 20% participant dropout. The spreadsheet with listed interventions was not accessible to other researchers until completion of the protocol. Enrolled participants received boxes 1 to 80 in order of enrollment and used the alphanumeric box codes for identification. Participants were asked to instill one drop of the given AT in both eyes three times daily for seven days, spaced evenly throughout the day, and to return for follow-up on the seventh day. PEG-ATs were chosen as the control because of their similar physical properties and vial appearance, because saline solution has a noticeably lower viscosity than CMC-ATs.^26^ Manufacturer markings were removed from all vials.

Primary outcomes included alpha diversity, beta diversity, and relative abundance of bacterial genera derived from pooled conjunctival swabs at days 1 and 7 for each participant. Secondary outcomes included the OSDI survey and compliance scores, collected from a computerized survey. Compliance scores were self-reported by participants as the estimated number of AT drops used in each eye daily, with ideal scores at one drops per eye daily although participants may have used more or fewer drops. The OSDI is a validated survey for assessing subjective dry eye symptomatology and quality of life, ranging from a score of 0 indicating no symptoms up to 48 indicating severe symptoms.

### Ocular Surface Microbiome Sample Collection and Processing

Before each conjunctival swab, one drop of proparacaine was instilled in both eyes. A sterile solid-core nylon swab (DNA/RNA Shield Swab collection kit; Zymo, Orange, CA, USA) was rotated in the inferior fornix of the left eye, then the right eye, and placed into a bead-beating tube containing 750 µL DNeasy lysis buffer (DNeasy PowerSoil Pro DNA extraction kit; Qiagen, Hilden, Germany) and stored at 4°C until further processing. Several blank swabs with the same proparacaine, swabs, and tubes were performed in room air without a participant present as controls.

Genomic DNA was extracted using the DNeasy PowerSoil Pro DNA extraction kit according to the manufacturer's instructions. For each sample, the V1-V3 hypervariable region of the 16S rRNA gene was amplified using primers 27F (5ʹ AGAGTTTGATCCTGGCTCAG 3ʹ) and 534R (5ʹ ATTACCGCGGCTGCTGG 3ʹ) with barcodes to allow multiplex deep sequencing as described previously.^36^ Each 20 µL polymerase chain reaction (PCR) mixture contained 2 µL of extracted DNA, 100 nM of the forward primer, 100 nM of the reverse primer, and 0.15 µL of Accuprime Taq High Fidelity Polymerase, 2 µl 10X PCR buffer II (Invitrogen, Carlsbad, CA, USA). Cycling conditions were as follows: denaturation at 95°C for two minutes, 25 cycles of denaturation at 95°C for 20 seconds, annealing at 56°C for 30 seconds and extension at 72°C for five minutes. PCR products were analyzed on 1% SYBR Safe agarose gel. Gel slices containing the amplicons were extracted and purified using the NucleoSpin Gel and PCR Clean-up kit (Machery-Nagel, Bethelhem, PA, USA). Purified PCR products were quantified using the Qubit HS DNA quantification kit (Invitrogen) and pooled in equimolar concentration. Quantitative PCR was used to quantify the concentration of the pooled PCR products using Library Quant Kit (Kapa Biosystems, Wilmington, MA, USA). The use of barcodes allowed multiplexing and bidirectional sequencing on the Illumina MiSeq platform (Illumina, San Diego, CA, USA).

### Data Processing and Statistical Analysis

Raw MiSeq reads (covering the V1–V3 hypervariable region of the 16S rRNA gene using primers 27F and 534R) were demultiplexed using cutadapt4.^37^ Demultiplexed reads were imported into Qiime2 v2021.11 and denoised by dada2 pipeline in qiime2.^38^ Taxonomy was assigned using the silva 138 SSU nr99 dataset and classify-sklearn.qiime, and the phylogeny plugin was used to generate unrooted and rooted trees. Taxa with greater than 10 reads identified in any negative control swabs were excluded from analysis and negative controls were compared to samples by UniFrac analysis. Qiime2 diversity plugin was used to perform core metrics analysis, alpha-group significance, and beta-group significance at the level of amplicon sequence variants (ASVs) with a 95% confidence interval. Classical statistics including analysis of variance (ANOVA), permutation multivariate analysis of variance (PERMANOVA), and Mann-Whitney tests were validated in SPSS at the same 95% confidence interval for the appropriate data type. Comparisons between timepoints were performed with pairwise assumptions. LEfSe analysis was performed at https://huttenhower.sph.harvard.edu/galaxy/.^39^

## Results

Among the 80 participants enrolled, 35 participants in the CMC group completed the follow-up period, and 31 participants in the PEG group completed the follow-up period. No intervention-associated complications were observed, and both interventions were well tolerated. Participant characteristics are displayed in the Table. Most participants were female (70%), did not wear contact lenses (77%), and had no history of ocular surgery (74%). Between-group analyses did not show any significant differences in participant characteristics. Among the participants who were enrolled and randomized (N = 80), 14 participants dropped out from the study either by not completing the survey upon follow-up, or by missing the follow-up visit altogether. Two participants later volunteered scheduling conflicts as the cause of dropout, whereas the remaining participants could not be reached. Participant dropout rates were also not significantly different between study arms (Fig. 1) by χ^2^ test (*P* = 0.239). Similar compliance was reported in both arms of the study, because most participants reported using two or three drops of ATs after being instructed to use three drops daily in both eyes. One participant in the CMC arm and two participants in the PEG arm, however, reported using no drops. Two participants in the CMC arm also reported using an average of five drops daily.

**Table.** Participant Characteristics

	Intervention Group		
Characteristic	CMC	PEG	*P* Value (Statistic)
Age, mean (SD)	41.2 (17.8)	40.0 (13.4)	0.760 (*t*)
Sex			0.256 (χ^2^)
Male	12	8	
Female	23	23	
Contact Lens Use			0.979 (χ^2^)
Yes	8	7	
No	27	24	
Ocular surgical history			0.454 (χ^2^)
Yes	7	10	
No	28	21	
Compliance (drops), mean (SD)	2.86 (0.81)	2.16 (0.82)	0.139 (U)

Participant characteristics were not significantly different between treatment groups at a 95% confidence threshold using statistical tests indicated (*t*, independent-samples; χ^2^, χ^2^ test of homogeneity; U, Mann-Whitney U).

**Figure 2.**
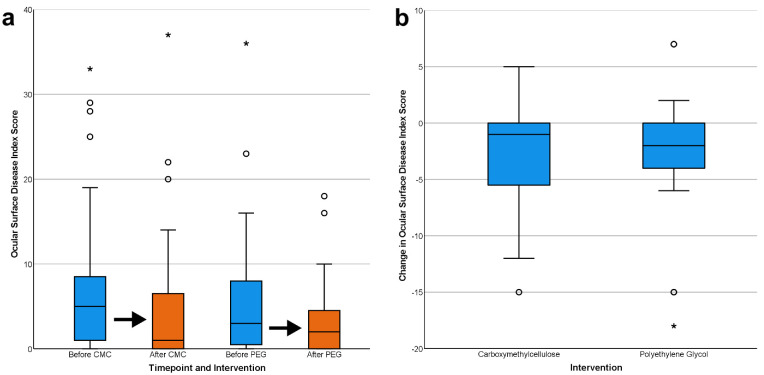
Box plots of ocular surface disease index scores. Outliers are shown as circles, and extreme outliers are shown as asterisks. (a) Ocular surface disease index scores are significantly lower after either CMC or PEG interventions. (b) There was no significant difference in reduction of ocular surface disease index scores between groups.

At baseline, the mean (standard deviation [SD]) OSDI scores were 7.69 (9.35) for the CMC group and 5.71 (7.70) for the PEG group. After intervention, the mean (SD) OSDI scores were 4.77 (7.93) for the CMC group and 3.35 (4.56) for the PEG group. This constituted a mean ΔOSDI (SD) of −2.91 (4.43) for the CMC group and −2.35 (5.42) for the PEG group. Both interventions were associated with a significant reduction in OSDI scores by pairwise Wilcoxon signed rank test (Fig. 2a, Z_CMC_ = −3.549, *P*_CMC_ < 0.01, Z_PEG_ = −2.390, *P*_PEG_ = 0.02), but there was no significant difference in ΔOSDI between interventions by Mann-Whitney U test (Fig. 2b, *U* = 590, *P* = 0.54).

**Figure 3.**
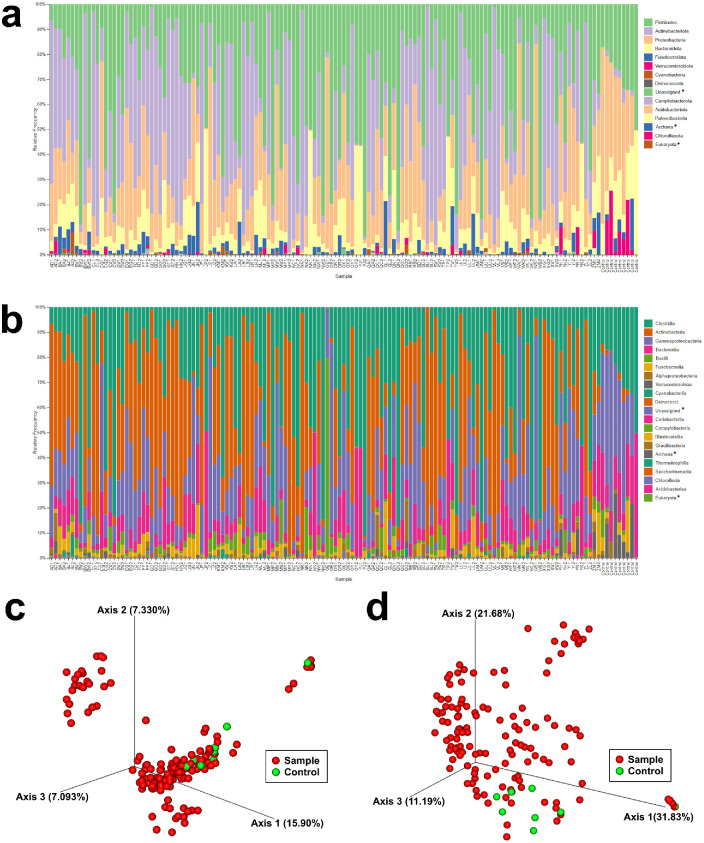
Next-Generation Sequencing Results. Taxonomic classification summary for all unfiltered samples at the (a) phylum and (b) class level. Sample letters indicate participant and numbers indicate before or after intervention. Asterisks indicate no classification available at this taxonomic level. (c) Principal coordinate analysis of unweighted UniFrac distances showing negative control samples have a distinct grouping within unfiltered sample microbiomes. (d) Principal coordinate analysis of weighted UniFrac distances showing negative control samples have a distinct grouping within unfiltered sample microbiomes.

**Figure 4.**
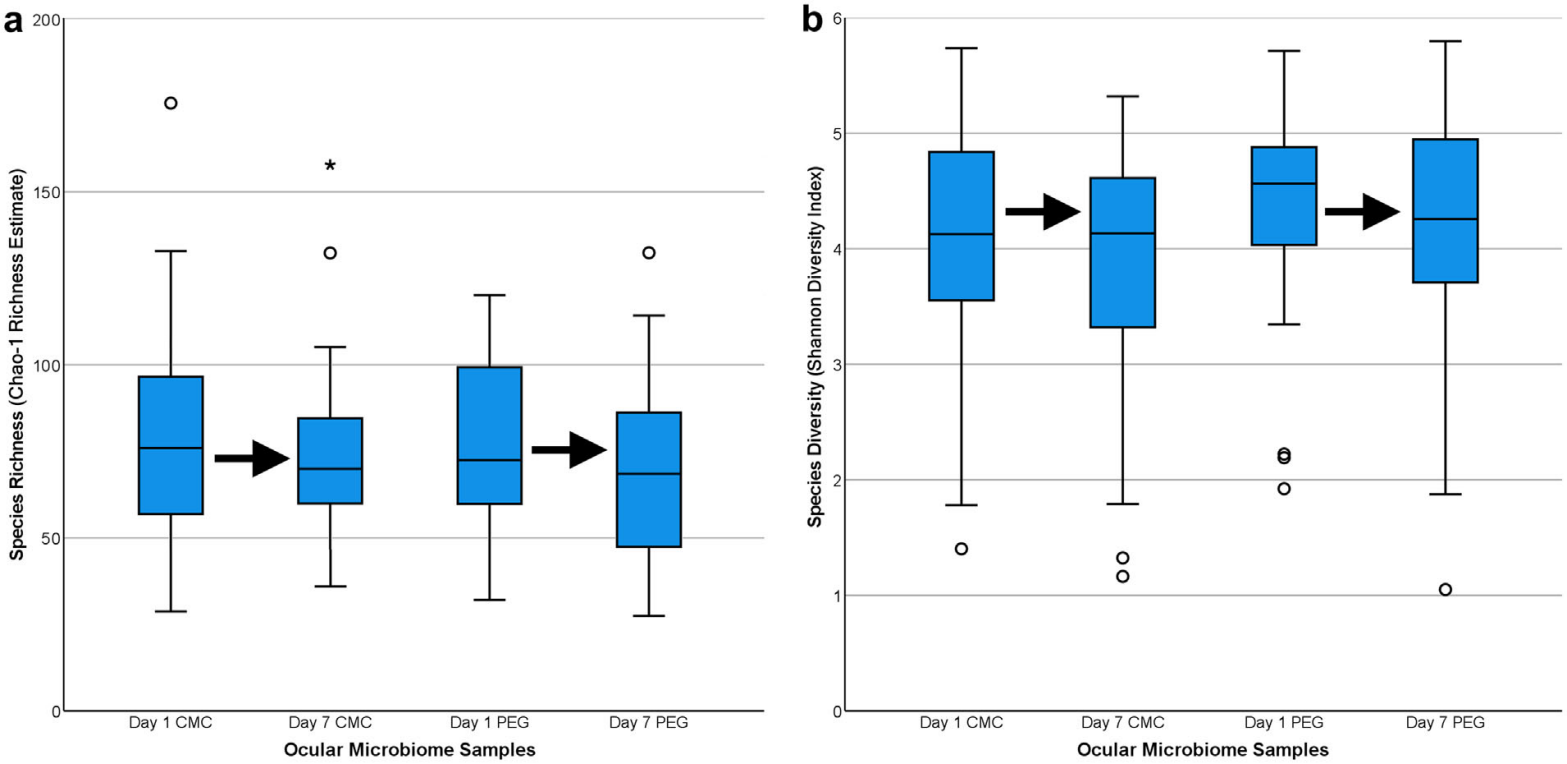
Box plots of alpha diversity. Alpha diversity measures are displayed for samples collected from both intervention groups at days 1 and 7. Outliers are shown as circles, and extreme outliers are shown as asterisks. (a) Species richness is not significantly different between groups at any time point. (b) Species diversity is not significantly different between groups at any time point.

Sequencing generated a maximum of 590 ASVs. The total number of reads from all participant samples and controls was 8,894,613 and was reduced to 4,554,231 after filtering and denoising. After contaminant filtering, 490 ASVs remained for analysis and were classified into 12 phyla (Fig. 3a), 18 classes (Fig. 3b), 53 orders, 100 families, and 236 genera. Beta diversity analysis (Figs. 3c, 3d) revealed grouping of controls. Species richness as measured by the Chao-1 estimate is displayed in Figure 4a. Between-group analyses showed no significant difference in richness between the CMC group and PEG group at any time and showed no change in richness after either intervention (ANOVA, *P* = 0.231). Species diversity as measured by the Shannon index are displayed in Figure 4b. Between-group analyses showed no significant difference in diversity between the CMC group and PEG group at any time and showed no change in diversity after either intervention (ANOVA, *P* = 0.224). Overall, neither intervention changed alpha diversity.

**Figure 5.**
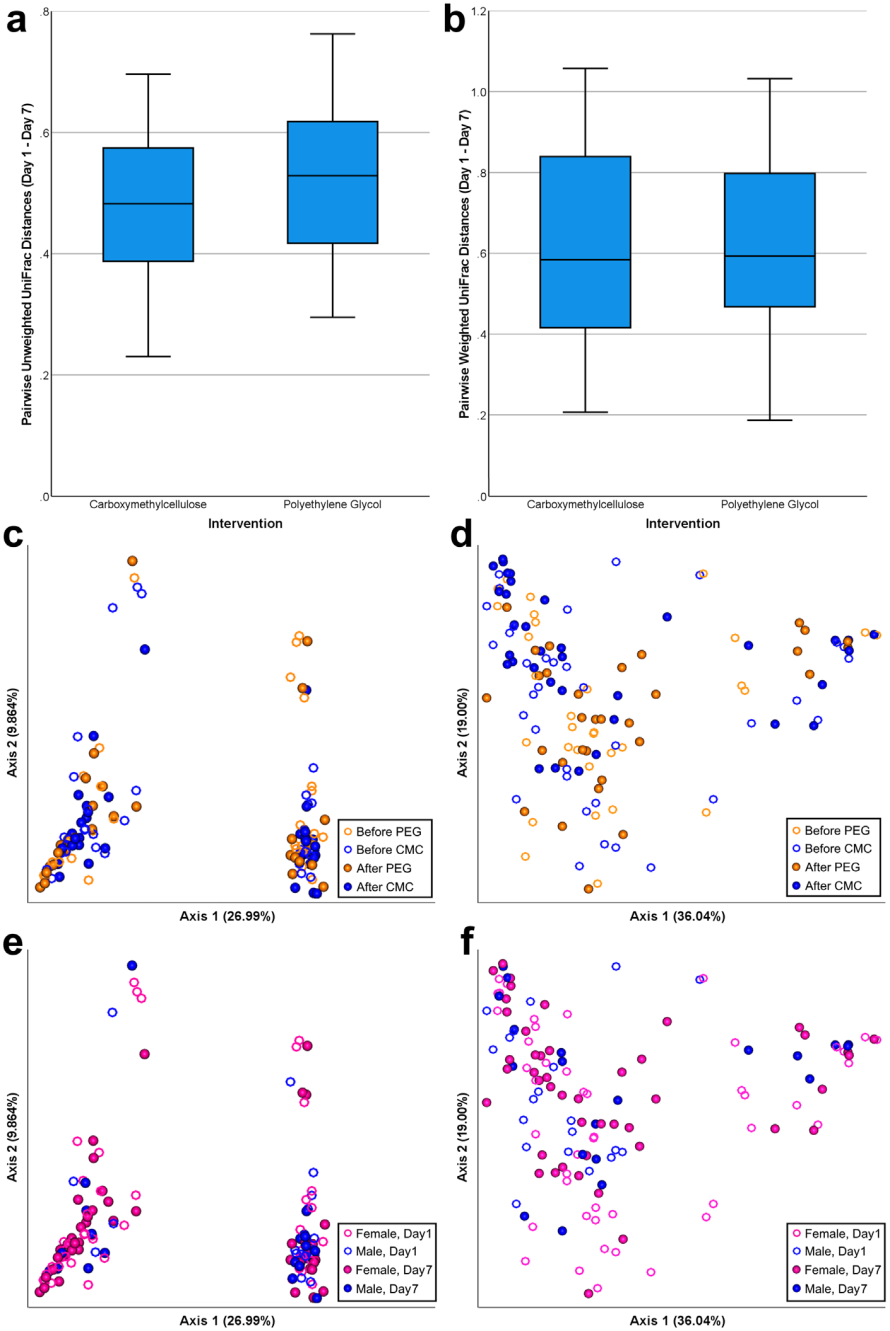
Beta diversity analysis. (a) Box plot of pairwise unweighted Unifrac distances between day 1 and day 7 time points, showing no significant differences between the two intervention groups. (b) Box plot of pairwise weighted UniFrac distances between day 1 and day 7 time points, showing no significant differences between the two intervention groups. (c) Principal coordinate analysis of unweighted UniFrac distances showing no significant grouping by intervention or time point. (d) Principal coordinate analysis of weighted UniFrac distances showing no significant grouping by intervention or time point. (e) Principal coordinate analysis of unweighted UniFrac distances shows grouping by participant sex. (f) Principal coordinate analysis of weighted UniFrac distances shows no significant grouping by participant sex.

**Figure 6.**
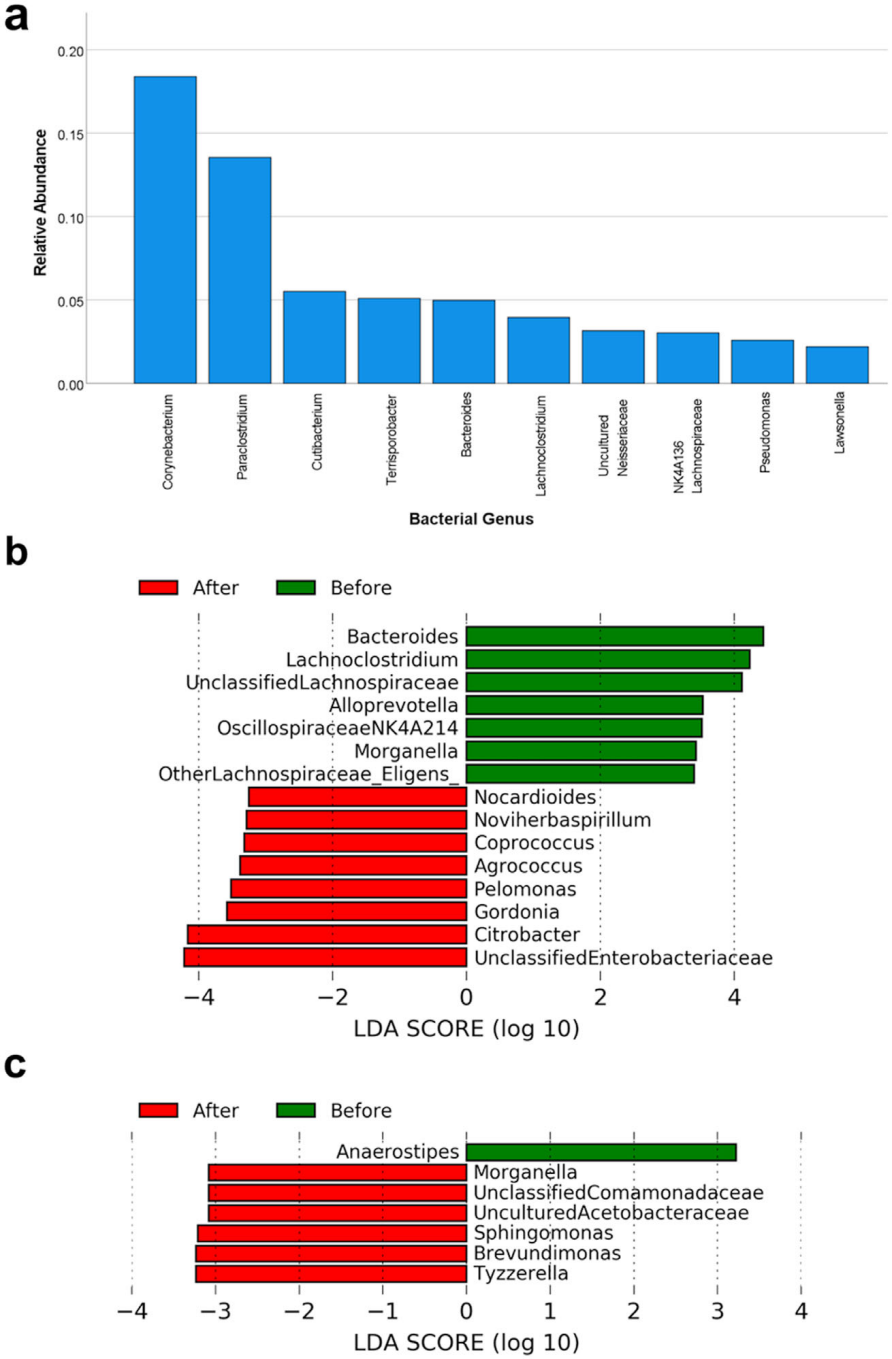
Bacterial relative abundance. (a) Bar graph of the top 10 most abundant bacterial genera from all ocular surface microbiome samples, with mean relative abundances listed. (b) LEfSe taxonomic analysis of taxa before and after CMC AT treatment at the genus level. (c) LEfSe taxonomic analysis of species before and after PEG AT treatment at the genus level.

Differences in the microbial community structure were compared using UniFrac. Mann-Whitney repeated-measure comparisons of unweighted UniFrac distances (*U* = 448, *P* = 0.227) and weighted UniFrac distances (*U* = 540, *P* = 0.9795) did not show any significant between-group differences (Figs. 5a, 5b). Samples did not separate by time point (PERMANOVA, *P* = 0.223) or intervention group (PERMANOVA, *P* = 0.668). However, separation was observed among samples along the first principal coordinate axis in unweighted UniFrac (Fig. 5c), but not in weighted UniFrac (Fig. 5d). Interestingly, samples clustered significantly by participant sex (PERMANOVA, *P* = 0.002) in unweighted UniFrac analysis (Fig. 5e), but this clustering was not observed in weighted UniFrac analysis (Fig. 5f). Although the overall microbiome community structures were not significantly altered by AT treatment, the relative abundance of several taxa was significantly changed by the treatments. Figure 6a shows the 10 most abundant bacterial genera from all OSM samples. LEfSe taxonomic analysis revealed that CMC treatment depleted several taxa including *Bacteroides* and *Lachnoclostridium*, and enriched *Enterobacteriaceae*, *Citrobacter*, and *Gordonia* (Fig. 6b). PEG treatment depleted *Anaerostipes* and enriched *Tyzzerella*, *Brevundimonas*, and *Sphingomonas* (Fig. 6c).

## Discussion

To our knowledge, this is the first randomized double-masked clinical trial assessing the effect of CMC-ATs on the OSM. Existing studies comparing AT formulations found minimal differences in subjective and objective outcomes among over-the-counter ATs but have yet to examine implications for the OSM.^40^ Although ingested CMC has been shown to affect the gut microbiome and DES has been associated with unique OSMs, there is no prospective evidence explicitly linking topical CMC to the OSM.^21^^,^^22^^,^^27^^,^^41^ One study found changes in the OSM after topical application of sodium hyaluronate, but only compared preserved and non-preserved solutions.^32^ Another study found no changes of the eyelid margin microbiome based on eyedrop preservatives.^42^ As a result, there are currently few reasons to choose one AT over another comparable formulation.^43^ To better understand how the OSM may mediate DES and AT therapy, we conducted this trial comparing the OSM effects of CMC-ATs with a control PEG-AT without CMC.

### Participants, Compliance, and Dry Eye Symptoms

Participants who completed the study were healthy adults without significant comorbidities, and there were no significant differences in the demographics of both study arms (Table). Compliance was also not statistically significantly different between the study arms and is unlikely to be a confounder.

On enrollment, both study arms had comparable DES symptomatology, with OSDI scores less than 22 in 60 participants (90.1%). Therefore only the remaining six participants were considered to have moderate or severe DES. Both study arms experienced a small and statistically similar reduction in OSDI (Fig. 2). These results are consistent with prior studies and suggest that CMC-ATs and PEG-ATs are comparable treatments for DES, although the effect size was smaller in this study than previously reported. A masked, randomized, multicenter trial estimated a −10 reduction in OSDI after seven days of CMC-ATs, but participants in that trial had greater DES symptoms at baseline (mean OSDI = 41).^44^

### OSM Diversity

When examining OSM diversity, several measures have been reported in the literature.^45^ Alpha diversity is a measure of microbiome diversity within a single sample, whereas beta diversity is a measure of diversity between samples. Comparisons with other studies should consider that this analysis used the Chao-1 species richness estimate^46^ and Shannon's diversity index^47^ as measures of alpha diversity whereas UniFrac^48^ was used as a measure of beta diversity.

Similar Chao-1 estimates and Shannon diversities between the CMC and PEG study arms and time points (Figs. 4a, 4b) indicate that neither CMC-AT nor PEG-AT interventions changed the alpha diversity of the OSMs. A randomized controlled study by Zhong et al.^32^ on 16 healthy adults also found no significant differences in the OSM alpha diversity after two weeks of sodium hyaluronate ATs with or without benzalkonium chloride preservative. Another randomized controlled study on 36 patients by Willis et al.^22^ found small increases in alpha diversity after one month of saline solution eye washes in DES patients. However, alpha diversity was already higher in DES patients than in healthy controls at baseline, and this change was not found in healthy controls after saline solution eye washes.

Overall, it appears that the high microbial diversity associated with DES may be resilient to topical elution or short-term interventions. Based on these two trials, it is possible that long-term AT use or saline elution exceeding one month may change alpha diversity, but there is no evidence that it may convert a DES-associated OSM into a healthy control-associated OSM by reducing alpha diversity. Some interventions such as topical prostaglandin therapy may increase alpha diversity,^29^ but other interventions such as contact lens wear do not.^20^ Successful treatment of DES may also reduce the alpha diversity, but none of these studies examined changes in DES symptomatology, and our study yielded only small reductions in DES symptomatology in healthy individuals, which do not represent successful treatment.

There were no significant differences in UniFrac distances between study arms (Figs. 5a, 5b) in this study, nor did principal coordinate analysis reveal any grouping related to AT use or study arm (Figs. 5c, 5d). Zhong et al.^32^ found distinct OSM beta diversities after two weeks of sodium hyaluronate ATs; their study was only controlled to compare preservatives in ATs and did not include controls using other ATs without sodium hyaluronate. Willis et al.^22^ did find unique beta diversities in DES patients, but no change associated with saline solution washes. Topical glaucoma medication and contact lens use, however, were associated with unique beta diversities in other studies. Overall, tear film elution does not appear to be associated with changes in OSM phylogenetic structure, but compounds such as sodium hyaluronate or behaviors such as contact lens wear change the taxa present on the conjunctiva. Therefore our study did not find that CMC or PEG are one of the compounds that affect beta diversity.

Among the variables examined in our study, gender explained the separation in microbiome beta diversity among samples (Fig. 5e). In the United States, females are more likely to use ocular cosmetics such as eyeliner and mascara, and these represent reservoirs or vectors for microbial contamination.^49^ Pigment accumulation is also present in the conjunctiva and lacrimal system of long-term cosmetic users, which may also alter the metabolism and substrate of the microbial community.^50^ Other studies, however, have not found associations between gender and beta diversity.^18^^,^^51^ Nevertheless, our study was not powered to control for gender differences, and thus the significance of this finding remains uncertain.

### OSM Composition

Before the advent of metagenomic analysis using bacterial ribosomal RNA high-throughput sequencing, the ocular surface was thought to have low bacterial abundance and diversity dominated by onfallen skin flora.^9^^,^^52^ Although conventional culture can reveal many of the bacterial species present in the OSM, metagenomic analysis has now revealed a community of low-abundance bacteria on the ocular surface which should be accounted for.^33^^,^^42^ The ocular surface has a relatively stable core OSM shared between most individuals, consisting of common genera such as *Corynebacterium, Staphylococcus, Streptococcus, Pseudomonas, Acinetobacter, and Propionibacterium*.^16^^,^^53^ Less commonly identified genera such as *Bacteroides* and *Neisseria* remain present in individuals after serial swabs, potentially representing an individual-specific core OSM as well. Other sporadically identified genera such as *Acidovorax* may be a result of contamination or are only transiently present on the ocular surface.

In our participants, we found high relative abundances of *Corynebacterium, Cutibacterium*, *Bacteroides, Pseudomonas*, and *Neisseriaceae* genera consistent with previous analyses of the core OSM (Fig. 6a). High *Cutibacterium* abundance may be related to our anesthetized swab methodology.^31^ Sporadically identified *Paraclostridium*, *Terrisporobacter, Lachnoclostridium,* and *Lachnospiraceae* genera in the phylum *Firmicutes,* as well as the genus *Lawsonella,* were also identified in our participants at a higher abundance than previously reported, although 16S ribosomal analysis in some studies used other hypervariable regions such as V4 and V8, which may have skewed abundances.^31^^,^^53^ Unusual anaerobic taxa such as *Paraclostridium* were present in the OSM consistent with other studies^53^ and may either reside in low-oxygen environments such as the inferior fornix, nasolacrimal duct, and meibomian glands. They may also have digestive tract origins and migrate to the eye through droplets or direct contact. Unifrac analysis (Figs. 3c, 3d) demonstrates that negative control swab samples had similar beta diversities and formed a distinct group. Therefore contamination is unlikely to account for the unusual but high-abundance taxa in Figure 6a.

To identify differentially abundant taxa between groups, we used LEfSe (Figs. 6b, 6c), a method for identifying statistically significant features that may explain differences between microbial samples. Treatment with CMC-ATs depleted *Bacteroides*, and no reduction was observed after PEG-ATs. In contrast, Zhong et al.^32^ found an increase of *Bacteroides* (phylum *Bacteroidetes*) after treatment of sodium hyaluronate ATs.^32^ Previous characterization of the OSM has found an association between *Bacteroidetes* enrichment and DES.^21^ Therefore treatment of DES with CMC-ATs may be related to *Bacteroides* depletion, although *Bacteroides* only represents one genus and not all of the *Bacteroidetes* phylum.

CMC-ATs were associated with enrichment of periocular skin-associated phyla *Actinobacteriota* (*Nocardioides, Agrococcus, Gordonia*) and depletion of *Firmicutes* (*Lachnoslostridium* and other *Lachnospiraceae* genera) except for *Coprococcus* (phylum *Firmicutes*), which was enriched by CMC-AT treatment. Conversely, *Actinobacteriota* depletion and *Firmicutes* enrichment has been associated with meibomian gland dysfunction, so these phyla may also be markers for therapeutic response to CMC-ATs in DES.^54^ PEG-ATs, however, had conflicting results at the *Firmicutes* phylum level, as *Anaerostipes* was depleted by PEG-AT treatment while *Tyzzerella* was enriched. It remains to be seen whether *Firmicutes* genera represent useful markers for DES and AT treatment or if these are findings specific to our population.

Both CMC-AT and PEG-AT treatments were associated with enrichment of *Proteobacteria* genera (*Noviherbaspirillum, Pelomonas, Sphingomonas, Brevundimonas*, and *Citrobacter*) and genera among other bacterial families (*Enterobacteriaceae, Comamonadaceae,* and *Acetobacteraceae*). PEG-ATs also enriched the genus *Morganella*, but CMC-ATs had the opposite effect by depleting *Morganella*. Although DES has been associated with *Proteobacteria* depletion,^21^ our participants overall experienced small improvements in DES symptoms after either AT intervention. The core genus whose depletion is associated with DES, *Pseudomonas,* was also unaffected by either AT treatment in our study. Instead, enrichment of *Proteobacteria* at the follow-up visit may be a result of greater swab depth, after the participants or investigators became more familiar and comfortable with the procedure.^12^

Overall, CMC-ATs were associated with enrichment of *Bacteriodota* and *Actinobacteriota*, and possibly depletion of *Firmicutes* as well. PEG-ATs were not associated with any of these changes, but both AT treatments resulted in *Proteobacteria* enrichment. Although CMC-ATs appeared to shift the OSM away from DES and toward a normal eye, it should be noted that this study was conducted on generally healthy patients without significant DES, and both AT arms experienced comparable reductions in DES symptomatology. Only one week was given as a washout period, which may not have been sufficient to revert an OSM to wild type. Furthermore, swabs were collected in 2022, when mask-wearing was mandatory in clinical spaces because of the COVID pandemic, which may have influenced the OSM.^55^^,^^56^ Interestingly, *Nocardioides* and *Pseudomonas* genera were detected, but neither pathogenic *Nocardia* nor *Pseudomonas aeruginosa* were identified in any participant, and no ocular infections were reported in our patient population.

### Study Limitations

Interpretation of this study should consider that AT compliance was participant-reported and not actively monitored (i.e., checking empty vials) as other studies have done.^57^ In the real-world setting, patients generally do not reliably adhere to a strict AT regimen, so a modified intention-to-treat analysis was chosen for this study, wherein only participants who missed the follow-up survey or follow-up swab were excluded from the analysis.^58^ Considering that few of our participants had DES and OSDI reductions were small, the Hawthorne effect may also have resulted in study bias.^59^ Our study also had no objective measures for DES such as Schirmer's test, tear breakup time, or tear osmolarity to confirm reduction of DES signs.

Although the objective of this study was to identify effects of CMC on the OSM, neither pure CMC solution nor a perfect control were available. Instead, commercially available ATs were chosen, with different inactive ingredients such as ionic salt buffers, organic (Aminomethylpropanol) salt buffers, hyaluronate, and sorbitol. Although these differences may confound results, their concentration is relatively low. Participants were also compared by time point with no differences in OSM diversity, suggesting that the impacts of these ingredients are not significant confounding factors.

Interpretation of these microbiome results should consider that this was a bilateral pooled, anesthetized, and deep conjunctival swab with nylon flocking. The bacterial microbiome at the lid margin may be different from the inferior fornix, and samples may also be affected by anesthesia and swab type.^31^^,^^60^ Although samples could be collected with a softer swab and without anesthesia, participants may not tolerate full-depth swabs reliably. Analysis should also consider the segment of 16S ribosome chosen (V1-V3), because the region chosen may bias bacterial relative abundances.^53^

## Conclusion

In this double-masked randomized controlled trial, CMC AT treatment increased *Bacteroides* and *Actinobacteriota* but decreased *Firmicutes* bacteria in the OSM of healthy adults, compared to control PEG AT treatment. Bacterial genera from a core OSM were identified but were unaffected by AT treatment. Neither CMC nor PEG ATs changed the OSM diversity, although both treatments resulted in small improvements in dry eye symptoms. These results suggest that CMC-induced changes in the OSM at the genus level may mediate improvements in dry eye symptoms, but CMC ATs are not superior to PEG ATs for the treatment of mild DES.
